# Are There Maternal Deaths Related to Hemorrhagic Stroke Due to Hypertensive Disorder of Pregnancy That Could Be Potentially Preventable by Tight Hypertension Management in Antepartum? A Retrospective Study from the Maternal Death Exploratory Committee in Japan

**DOI:** 10.3390/jcm12082908

**Published:** 2023-04-17

**Authors:** Hiroaki Tanaka, Junichi Hasegawa, Shinji Katsuragi, Kayo Tanaka, Tatsuya Arakaki, Masamitsu Nakamura, Eijiro Hayata, Masahiko Nakata, Akihiko Sekizawa, Isamu Ishiwata, Tomoaki Ikeda

**Affiliations:** 1 Department of Obstetrics and Gynecology, Mie University School of Medicine, Edobashi 2-174, Tsu 514-8507, Mie, Japan; 2 Japan Maternal Death Exploratory Committee, Ichigayahachimantyou 14, Shinjuku, Tokyo 162-0844, Japan

**Keywords:** maternal death, hemorrhagic stroke, CHIPS randomized controlled trial

## Abstract

Background: Unlike Europe and the United States, Japan has seen numerous maternal deaths from hemorrhagic strokes related to hypertensive disorders of pregnancy (HDP). This study retrospectively analyzed deaths associated with HDP-related hemorrhagic stroke in Japan to determine the number of deaths that may have been prevented with blood pressure control during pregnancy. Methods: This study included maternal deaths related to hemorrhagic stroke cases. The proportion of patients without proteinuria whose blood pressure exceeded 140/90 mmHg between 14+0 and 33+6 weeks of gestation were determined. Lastly, the application of tight antihypertensive management was evaluated. Results: Among 34 HDP-related maternal deaths, 4 cases involved patients without proteinuria whose blood pressures exceeded 140/90 mmHg between 14+0 and 33+6 weeks of gestation. These included two chronic hypertension and two gestational hypertension cases. None of the patients received antihypertensive agents, and their blood pressures were managed leniently. Conclusion: Among HDP-related hemorrhagic stroke deaths in Japan, only a few cases of maternal death could have been prevented with tight blood pressure management, as described in the CHIPS randomized controlled trial. Therefore, to prevent HDP-related hemorrhagic stroke in Japan, new preventive strategies during pregnancy should be established.

## 1. Introduction

The management of non-severe pregnancy hypertension has been controversial. The 2016 CHIPS randomized controlled trial showed that for non-severe pregnancy hypertension, controlling the diastolic blood pressure (BP) to a target of 85 mmHg reduced various adverse maternal and perinatal outcomes [1]. Based on this, 140/90 mmHg became the standard threshold for initiating antihypertensive agents during pregnancy. The International Society for the Study of Hypertension in Pregnancy (ISSHP) recommended 135/85 mmHg as the BP target. Meanwhile, the National Institute for Health and Care Excellence recommended controlling the diastolic BP to 85 mmHg [2,3].

The CHIPS randomized controlled trial reported more maternal deaths, and serious complications, including stroke, were observed in severe hypertension or preeclampsia patients whose BPs were controlled leniently. 

In Western European countries, infarct strokes occur more frequently than hemorrhagic strokes during pregnancy [4]. However, in Japan, hemorrhagic strokes were more common than infarct strokes. Maternal deaths due to hemorrhagic stroke, associated with hypertension disorder of pregnancy (HDP), have become a problem [5,6,7]. Numerous pregnant women who died from HDP-related hemorrhagic strokes had developed a severe hemorrhagic stroke at the onset. After the hemorrhagic stroke, it was difficult to save most of the patients [7]. Therefore, preventing the onset of hemorrhagic stroke is important for reducing maternal deaths. We hypothesized that maternal deaths associated with hemorrhagic strokes are due to bleeding from pre-existing persistent hypertension from chronic hypertension (CH) or gestational hypertension (GH), and that the tight control of these non-severe or severe hypertensions may reduce hemorrhagic strokes. This study retrospectively analyzed maternal deaths from HDP-related hemorrhagic stroke in Japan and determined the number of non-severe or severe pregnancy hypertension cases that should have been managed with tight BP control (diastolic BP < 85 mmHg), according to the ISSHP standards.

## 2. Materials and Methods

The number of maternal deaths caused by HDP-related hemorrhagic stroke in Japan from 2010 to 2020 were extracted from the Japan Maternal Death Exploratory Committee of the Japan Association of Obstetricians and Gynecologists. 

### 2.1. Diagnosis of Hemorrhagic Stroke and HDP

Hemorrhagic stroke cases diagnosed by autopsy or computed tomography were included. In cases involving HDP, hemorrhagic strokes due to cerebral arteriovenous malformations or moyamoya disease were excluded. 

HDP was diagnosed and classified based on the ISSHP classification. This study included hemorrhagic strokes associated with HELLP syndrome. HELLP syndrome was diagnosed based on the Mississippi classification proposed by Sibai et al. [8,9]. 

### 2.2. Patient Characteristics

The following patient characteristics were surveyed: age, primipara, body mass index >25, the timing of hemorrhagic stroke (antepartum, intrapartum or during C-section, and postpartum), gestational weeks of delivery, mode of delivery (C-section, vaginal delivery, and undelivery), neurosurgery, maternal complications without hypertension, and obstetrics complications without HDP. 

### 2.3. Proportion of Maternal Deaths Caused by Hemorrhagic Stroke Due to CH and GH with and without Antihypertensive Medication

We investigated maternal deaths related to hemorrhagic stroke due to HDP. Among these, we selected hemorrhagic strokes related to patients without proteinuria and whose BP exceeded 140/90 mmHg between 14+0 and 33+6 weeks of gestation for hemorrhagic strokes related to CH and GH. We investigated whether antihypertensive medication was given according to the protocol of the CHIPS study.

### 2.4. Time from BP Elevation (>140/90 mmHg) to Cerebral Hemorrhage

In all patients who died of HDP-related hemorrhagic stroke, the time from the first time blood pressure exceeded 140 mmHg systolic BP or 90 mmHg diastolic BP to cerebral hemorrhage was investigated.

## 3. Results

From 2010 to 2020, there were 486 maternal deaths in Japan. Among these, 34 patients (7%) died from HDP-related hemorrhagic strokes. 

### 3.1. Maternal Background

Table 1 shows the maternal characteristics. Their median age was 34 (23–45) years with 16 women (47%) aged 35 or older and seven women (21%) aged 40 or older. Half of the patients were primiparous. Hemorrhagic stroke most commonly occurs during the intrapartum period or during the C-section. More than half of the deliveries were at term, and most were delivered by 34 weeks or later. Undelivery was defined as the gestational age when fetal death was confirmed. There were more C-sections than vaginal deliveries, and there were two cases (6%) of undelivery. Craniotomy for hematoma removal or neurosurgery for external ventricular drainage was performed in about half of the cases. Few maternal complications without hypertension were observed. In terms of obstetric complications without HDP, there were two cases of fetal growth restriction (6%), one case of threatened premature labor (3%), and one case of gestational diabetes mellitus (3%).

### 3.2. Proportion of Maternal Deaths Caused by Hemorrhagic Stroke Due to CH and GH with and without Antihypertensive Medication

**Figure 1:**
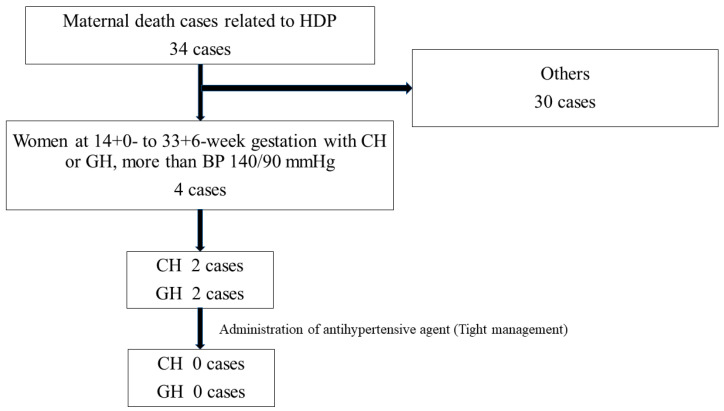
Patients without proteinuria and whose BP exceeded 140/90 mmHg between 14+0 and 33+6 weeks of gestation (CH and GH).

Out of 34 deaths from HDP-related hemorrhagic strokes, 4 cases involved patients without proteinuria and whose BP exceeded 140/90 mmHg between 14+0 and 33+6 weeks of gestation (Figure 1). These included two chronic hypertension (CH) and two gestational hypertension (GH) cases. None of the patients received antihypertensive agents during pregnancy, and their BP were leniently managed.

### 3.3. Time from BP Elevation (>140/90 mmHg) to Cerebral Hemorrhage

**Figure 2:**
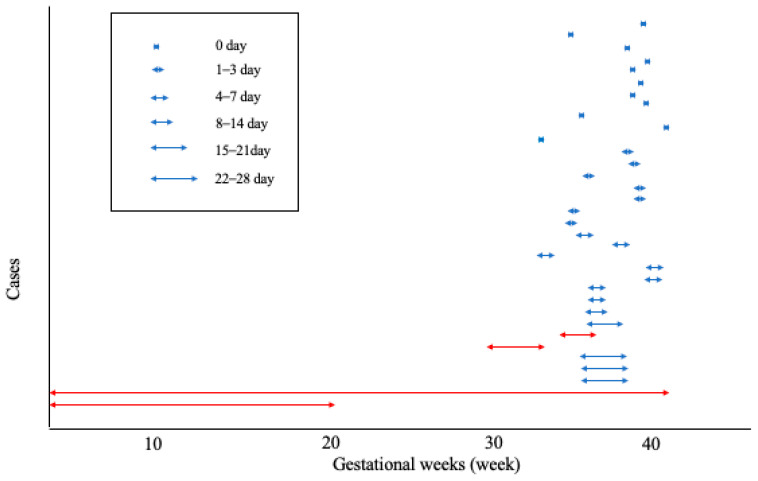
Duration of hemorrhagic stroke after blood pressure > 140/90 mmHg (red line indicates pregnant women with chronic hypertension or gestational hypertension whose blood pressure was greater than 140/90 mmHg).

**Figure 3:**
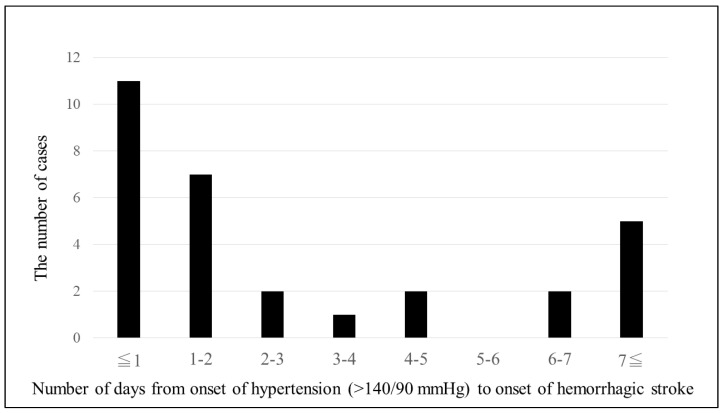
Time from BP elevation (>140/90 mmHg) to cerebral hemorrhage in 30 patients with proteinuria whose BP did exceed 140/90 mmHg between 14+0 and 33+6 weeks of gestation and whose BP did not exceed 140/90 mmHg between 14+0 and 33+6 weeks of gestation.

Figure 2 shows the duration of hemorrhagic stroke after a BP >140/90 mmHg. Figure 3 documents the first time a BP > 140/90 mmHg was detected before the onset of hemorrhagic stroke in 30 patients with proteinuria whose BP did exceed 140/90 mmHg between 14+0 and 33+6 weeks of gestation and whose BP did not exceed 140/90 mmHg between 14+0 and 33+6 weeks of gestation. In 11 cases, this occurred within 24 h of the hemorrhagic stroke (36%). In 18 cases, it occurred within 3 days (60%). In 25 cases (83%), it occurred within 1 week.

## 4. Discussion

This study emphasized three important findings. Firstly, most deaths from HDP-related hemorrhagic stroke involved patients with late-onset HDP. Secondly, about 12% (CH and GH) of the hemorrhagic strokes may have been prevented with tight management, in accordance with the ISSHP target (diastolic BP < 85 mmHg). Third, about one-third of maternal deaths from cerebral hemorrhage related to HDP exceeded 140/90 mmHg within 24 h of the onset of hemorrhagic stroke, and about one-third exceeded it within 3 days. 

HDP-related hemorrhagic strokes were more frequently observed in late-onset HDP patients than early-onset HDP patients. Differences have been reported between the early-onset and late-onset types [10,11]. The late-onset type was characterized by normal trophoblast infiltration of the spiral artery. Previously reported etiologies of the late-onset type include increased angiotensin II type 1 receptor sensitivity. However, this did not explain the higher incidence of hemorrhagic strokes [12]. Prior to starting this study, maternal deaths associated with hemorrhagic stroke were hypothesized to involve hemorrhages due to persistent hypertension from preexisting CH or GH. Based on this, strict BP control, described by the ISSHP, was indicated in pregnant patients developing hypertension to reduce maternal deaths associated with hemorrhagic stroke. The actual study results differed from the hypothesis as only a few cases involved persistent hypertension before the onset of hemorrhagic stroke. Most patients developed cerebral hemorrhage shortly after the onset of hypertension within a week of the hemorrhagic stroke. This implied that HDP-related hemorrhagic stroke involved late-onset HDP and followed an acute course. Early-onset HDP is due to chronic stress caused by placental hypoplasia [13]. Unlike in early-onset HDP, the acute stress in late-onset HDP reportedly induced syncytiotrophoblast stress, damaging the vascular endothelium [13]. Cerebral blood vessels are vulnerable to acute vascular endothelial damage and BP alterations. These are likely reasons for the higher incidence of hemorrhagic stroke in late-onset HDP. 

The RE-LY trial found that, in addition to age, stroke history, and aspirin or warfarin administration, being non-Caucasian was associated with hemorrhagic stroke [13]. Other studies have found that, in addition to hypertension, smoking, and excessive alcohol intake, being East Asian was also associated with hemorrhagic stroke [14,15]. Studies from Japan and China have also shown that HDP in Asians more frequently results in hemorrhagic stroke than infarct strokes [16,17,18]. Thus, HDP-related hemorrhagic strokes should be addressed specifically among Asian patients. 

There are only a few cases in which HDP-related hemorrhagic stroke has been reduced by tight BP management. This study considered no surviving cases. Additionally, we did not observe all pregnant women with hypertension. Therefore, we are not stating that tight BP management is meaningless. In Japan, pregnancy is publicly subsidized, and checkups are available every 1–4 weeks. Most women who become pregnant always see a health care provider in the early stages of pregnancy. It is hypothesized that tight BP management is less common in maternal deaths due to HDP-related hemorrhagic stroke. We would like to emphasize that new preventive strategies, in addition to tight BP management, are needed to further reduce maternal deaths from HDP-related hemorrhagic stroke in Japan. 

The number of maternal deaths In Japan has been decreasing year by year. Most of these deaths are due to obstetric hemorrhage. The issues that must be addressed in the future are hemorrhagic stroke, pulmonary thromboembolism, amniotic fluid embolism, and suicide. This study focused on hemorrhagic stroke. Until now, only deaths from hemorrhagic stroke have been registered in Japan. In Japan, the enrolment of surviving cases will begin in 2021. Once these cases are accumulated, new prevention strategies can be considered.

This study was limited due to its retrospective nature. The sample size was small because only deaths from HDP-related hemorrhagic stroke were analyzed.

## 5. Conclusions

In conclusion, most HDP-related hemorrhagic strokes in Japan involved late-onset HDP patients. Only a few cases of hemorrhagic strokes may have been prevented with tight BP management in cases of maternal death, according to the protocol in the CHIPS randomized controlled trial. Therefore, to prevent HDP-related hemorrhagic stroke in Japan, new preventive strategies during pregnancy should be established. 

## Figures and Tables

**Table 1:** Maternal background.

	n = 34
Age	34 (23–45)
Primipara	17 (50%)
BMI > 25	8 (24%)
Timing of hemorrhagic stroke	
Antepartum	10 (29%)
Intrapartum or during C-section	16 (47%)
Postpartum	8 (24%)
Gestational of weeks of delivery	
<32 gestational weeks	3 (9%)
32–36 gestational weeks	11 (32%)
>36 gestational weeks	20 (59%)
Mode of delivery	
C-section	20 (59%)
Vaginal delivery	12 (35%)
Undelivery	2 (6%)
Neurosurgery	16 (47%)
Maternal complication without hypertension	
Hyperthyroidism	1 (3%)
Hypothyroidism	1 (3%)
Obstetrics complication without HDP	
FGR	2 (6%)
Threatened premature labor	1 (3%)
GDM	1 (3%)

HDP, Hypertension disorder of pregnancy; FGR, fetal growth restriction; GDM, Gestational diabetes mellitus.

## Data Availability

The data that support the findings of this study are available from the corresponding author upon reasonable request.
